# Association of Salivary Human Papillomavirus Infection and Oral and Oropharyngeal Cancer: A Meta-Analysis

**DOI:** 10.3390/jcm9051305

**Published:** 2020-05-01

**Authors:** Óscar Rapado-González, Cristina Martínez-Reglero, Ángel Salgado-Barreira, Almudena Rodríguez-Fernández, Santiago Aguín-Losada, Luis León-Mateos, Laura Muinelo-Romay, Rafael López-López, María Mercedes Suarez-Cunqueiro

**Affiliations:** 1 Department of Surgery and Medical-Surgical Specialties, Medicine and Dentistry School, Universidade de Santiago de Compostela (USC), 15782 Santiago de Compostela, Spain; oscar.rapado@rai.usc.es; 2 Liquid Biopsy Analysis Unit, Translational Medical Oncology (Oncomet), Health Research Institute of Santiago (IDIS), 15706 Santiago de Compostela, Spain; lmuirom@gmail.com; 3 Centro de Investigación Biomédica en Red en Cáncer (CIBERONC), Instituto de Salud Carlos III, 28029 Madrid, Spain; 4 Methodology and Statistics Unit, Galicia Sur Health Research Institute (IISGS), 36312 Vigo, Spain; cristina.martinez@iisgaliciasur.es (C.M.-R.); angel.salgado.barreira@sergas.es (Á.S.-B.); 5 Department of Preventive and Public Health, Universidade de Santiago de Compostela (USC), 15782 Santiago de Compostela, Spain; almudena.rodríguez@usc.es; 6 Translational Medical Oncology (Oncomet), Health Research Institute of Santiago (IDIS), Complexo Hospitalario Universitario de Santiago de Compostela (SERGAS), 15706 Santiago de Compostela, Spain; lanfear22@hotmail.com (S.A.-L.); Luis.Angel.Leon.Mateos@sergas.es (L.L.-M.); 7 Translational Medical Oncology (Oncomet), Health Research Institute of Santiago (IDIS), 15706 Santiago de Compostela, Spain

**Keywords:** human papillomavirus, oral cancer, oropharyngeal cancer, saliva, meta-analysis

## Abstract

Background. Human papillomavirus (HPV) infection has been recognized as an important risk factor in cancer. The purpose of this systematic review and meta-analysis was to determine the prevalence and effect size of association between salivary HPV DNA and the risk of developing oral and oropharyngeal cancer. Methods. A systematic literature search of PubMed, EMBASE, Web of Science, LILACS, Scopus and the Cochrane Library was performed, without language restrictions or specified start date. Pooled data were analyzed by calculating odds ratios (ORs) and 95% confidence intervals (CIs). Quality assessment was performed using the Newcastle–Ottawa Scale (NOS). Results. A total of 1672 studies were screened and 14 met inclusion criteria for the meta-analysis. The overall prevalence of salivary HPV DNA for oral and oropharyngeal carcinoma was 43.2%, and the prevalence of salivary HPV16 genotype was 27.5%. Pooled results showed a significant association between salivary HPV and oral and oropharyngeal cancer (OR = 4.94; 2.82−8.67), oral cancer (OR = 2.58; 1.67−3.99) and oropharyngeal cancer (OR = 17.71; 6.42−48.84). Significant associations were also found between salivary HPV16 and oral and oropharyngeal cancer (OR = 10.07; 3.65−27.82), oral cancer (OR = 2.95; 1.23−7.08) and oropharyngeal cancer (OR = 38.50; 22.43−66.07). Conclusions. Our meta-analysis demonstrated the association between salivary HPV infection and the incidence of oral and oropharyngeal cancer indicating its value as a predictive indicator.

## 1. Introduction

Human papillomavirus (HPV) infection has been recognized as an important risk factor in a subset of head and neck squamous cell carcinomas, independently of traditional risk factors such as tobacco or alcohol use [1,2]. Globally, around 38,000 cases of head and neck cancer are attributed to the HPV infection. Of these, around 76% are cases of oropharynx cancer, 12% of oral cavity cancer and 10% of larynx cancer [3]. Currently, it is well known that HPV-status determines the molecular landscape of these tumors and their clinical evolution, with a better prognosis and response to therapy being found in HPV-positive patients [4,5].

HPVs are small, non-enveloped, close-circular, double-stranded DNA viruses of approximately 8000 base-pairs which present a specific tissue tropism infecting epithelial cells of the skin and mucosae of the anogenital and upper aero-digestive tract [6]. More than 200 different HPV types have been identified and classified into low-risk and high-risk according to their oncogenic potential. In this sense, high-risk HPV (HR-HPV) can promote the malignant transformation of HPV-infected cells through E6 and E7 viral oncoproteins, responsible for inactivating the *TP53* and *Rb* (retinoblastoma tumor suppressor gene) [7]. A subset of 12 alpha HR-HPV (16, 18, 31, 33, 35, 39, 45, 51, 52, 56, 58, and 59) has been classified as carcinogenic to humans according to the International Agency of Research in Cancer [8]. HR-HPV is considered the main cause of cervical cancer, genotypes 16 and 18 being responsible for 70% of cases [9]. In addition, several studies have also demonstrated the pathogenic role of HPV in other anogenital cancers [10,11,12] as well as in head and neck cancers [13]. Currently, HPV16 is widely recognized as an etiological factor in oropharynx tumors [14], however, not enough evidence exists regarding the HPV relationship and the anatomic subsites of head and neck squamous cell carcinoma [15]. 

Nowadays, a variety of molecular biological methods have been developed for the detection and genotyping of HPV at DNA, mRNA, and protein levels by polymerase chain reaction (PCR), real-time PCR, in situ hybridization, immunohistochemistry and serum antibody assays [16]. In addition, next-generation HPV sequencing approaches provide accurate information on genotype composition and pathways to better understand functional consequences [17]. Certain collection approaches present difficulties. For example, tumoral tissue biopsy is invasive and tumors may be inaccessible. For its part, the collection of oral exfoliated cells with cotton swabs or cytobrush is restricted to a specific and accessible oral area, making collection difficult for non-visual tumors and early molecular alterations. To overcome these drawbacks, the detection of HPV in oral exfoliated cells from saliva (with or without oral rinses) represents a quick and easy non-invasive alternative for oral and oropharyngeal cancer screening in high-risk populations. In this sense, several researchers have analyzed the prevalence of salivary HPV DNA from head and neck cancer, however, to our knowledge, no previous systematic review has elucidated evidence of this relationship. Therefore, the aim of the present systematic review and meta-analysis was to determine the prevalence and effect size of association between salivary HPV DNA and the risk of developing oral and oropharyngeal cancer. 

## 2. Materials and Methods

### 2.1. Protocol and Registration

This study was conducted according to Preferred Reporting Items for Systematic Reviews and Meta-Analysis (PRISMA) guidelines [18] and the protocol was registered with the International Prospective Register of Systematic Reviews (reference No. CRD42020161345).

### 2.2. Search Strategy and Study Selection

The systematic literature search was performed in PubMed, EMBASE, Web of Science, LILACS, Scopus and the Cochrane Library through 9 January 2020, without language restrictions or specified start date. The following combinations of keywords and medical subject headings were used: (human papilloma virus OR HPV) AND (saliva OR oral rinses OR mouthwash) AND (oral squamous cell carcinoma OR OSCC OR oropharyngeal squamous cell carcinoma OR OPSCC OR oral cancer OR oropharyngeal cancer). All studies were screened based on the title and abstract, and eligible manuscripts were retrieved for full-text review. Additionally, we manually searched the reference lists in each original and review article in order to avoid missing potential studies. The literature search was performed independently by two researchers (ORG and MMSC), and any disagreements were resolved by consensus. The studies selected through the search strategy and other references were managed using RefWorks software, and duplicated items were removed using the associated tools.

### 2.3. Eligibility Criteria

We included the studies that met the following criteria: (1) case-control studies of patients with oral and/or oropharyngeal cancer and healthy controls, (2) HPV DNA prevalence determined in salivary samples (whole saliva or oral rinses), and (3) sufficient data to calculate odds ratios (ORs) with 95% confidence intervals (CIs). The exclusion criteria were as follows: (1) in vitro or animal study, (2) reviews, letters, personal opinions, book chapters, case reports, and conference abstracts, and (3) duplicate articles or suspicion of data overlap.

### 2.4. Protocol and Registration

Two researchers (ORG and MMSC) independently assessed each eligible manuscript, extracted data using a pre-established form, and collated the data into a Microsoft Excel spreadsheet (Microsoft Corp. Redmond, WA, USA). Any disagreement among reviewers was resolved by consensus. The following information was extracted from each study: author, publication year, country, type of sample, method of collection, tumor location, sample size, HPV detection method, number of cases and HPV-positive cases, number of controls and HPV-positive controls, HPV-positive genotypes, overall HPV DNA prevalence (number of subjects testing positive for any HPV type) and type-specific HPV DNA prevalence (number of subjects testing positive for specific HPV types: HPV16 or HPV18, HR-HPV and LR-HPV). If the required data were incomplete, attempts were made to contact the authors to obtain the missing information.

### 2.5. Assessment of Risk Bias

The Newcastle-Ottawa Scale (NOS) [19] was used to evaluate the individual quality of the selected studies by three independent researchers (ORG, ARF, and MMSC), and discrepancies were resolved by consensus. The NOS assesses the quality of non-randomized studies based on design, content and ease of use directed to the task of incorporating the quality assessments in the interpretation of meta-analytic results. This ‘star system’ consists of 8 items classified into three broad perspectives: the selection of study groups; the comparability of the groups; and the ascertainment of either the exposure or outcome of interest for case-control or cohort studies. The highest quality studies were allotted a maximum of one star for each item, except for, the item related to comparability, which was allowed the assignment of a maximum of two stars. The NOS score ranged from 0 to 9 stars and validity criteria were as follows: 8–9, high quality; 6–7, medium quality; <5 low quality.

### 2.6. Statistical Analysis

Statistical analysis was conducted using the meta package of free R software (v.3.6.2; https://www.r-project.org). Firstly, to evaluate the statistical model applied to the meta analytic database, heterogeneity was assessed using the Cochran’s Q statistic test-based Chi-squared test and I2 statistics. Heterogeneity was considered significant when I2 > 50% and/or presence of a *p* < 0.10 for the Cochran’s Q test. The prevalence of HPV DNA and HPV genotypes in oral and/or oropharyngeal cancer was calculated using fixed or random effects depending on the heterogeneity. The relationship between saliva HPV DNA infection and oral and/oropharyngeal cancer risk was evaluated by pooled odds ratio (OR) and 95% confidence intervals (CIs) comparing cases to controls. If significant heterogeneity was detected, the DerSimonian and Laird random-effects model was applied to calculate the pooled OR with 95% CIs; otherwise, the Mantel–Haenszel fixed-effects model was used. Then, subgroup analyses were performed to explore the potential sources of heterogeneity among studies according to the anatomic tumor location and HPV genotypes. Additionally, publication bias was checked with Begg’s and Egger’s tests and by visual inspection in funnel plots demonstrating the relationship between the individual log ORs and their standard errors [20,21]. *p*-values of <0.05 were considered to indicate statistical significance. 

## 3. Results

### 3.1. Study Selection

**Figure 1:**
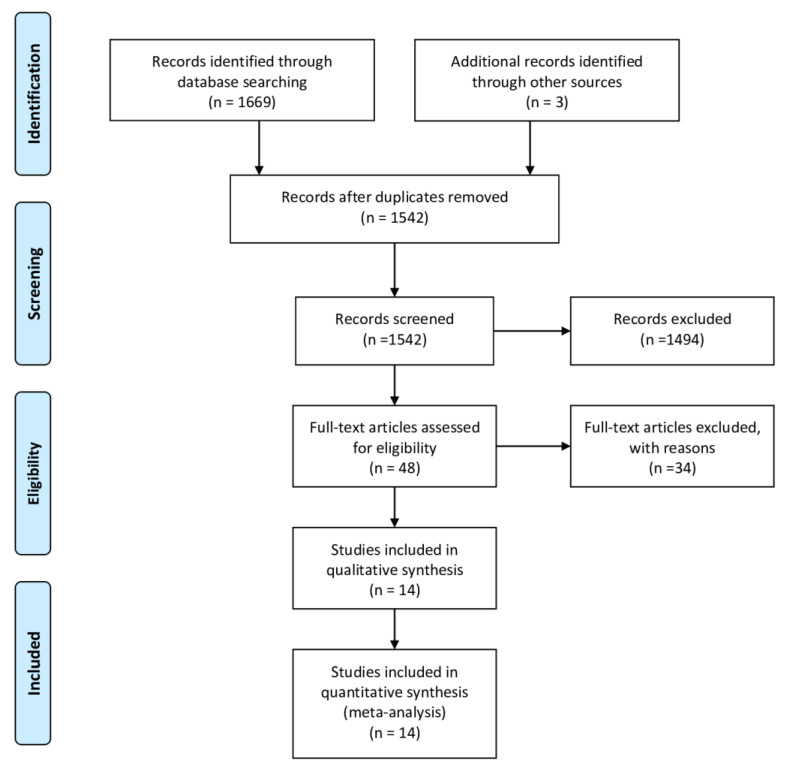
Preferred Reporting Items for Systematic Reviews and Meta-Analysis (PRISMA) flow diagram of the literature selection process, including identification, screening, eligibility and total studies included in qualitative and quantitative synthesis.

A total of 1669 articles were identified across the six electronic databases and three additional reports from the reference lists. After removing duplicates, a total of 1542 articles were screened based on the title and abstract, and 1494 were excluded for lack of adherence to our inclusion criteria. Therefore, full-text articles were retrieved for the remaining 48 articles. After a full-text review, 34 articles were excluded for the following reasons: non case-control studies (22); controls under risk conditions (2); suspicious of data overlap (3); insufficient data (3); and reviews, letters, and meta-analysis (4). Finally, 14 articles met all the inclusion criteria and were included in the final analysis. A detailed flowchart showing the selection process is shown in Figure 1.

### 3.2. Study Characteristics

Individual characteristics of the included studies are summarized in Table 1. A total of 14 articles evaluating HPV prevalence in oral and/or oropharyngeal cancer were included in this meta-analysis, and these studies were carried out from 2005 to 2019. Study sample sizes ranged from 42 to 677 subjects. The study units in this meta-analysis comprised a total of 2320 cases (658 from the oral cavity, 1160 from the oral cavity plus oropharynx and 502 from the oropharynx), and 5868 controls (2210 from the oral cavity, 2304 from the oral cavity plus oropharynx and 1354 from the oropharynx). As reported in Table 1, four studies were conducted in India [22,23,24,25], three in the USA [26,27,28], and two in Sweden [29,30], whereas the remaining studies were carried out in the following countries: Canada [31], France [32], Hungary [33], Pakistan [34], and Iran [35]. In terms of sampling, oral rinses and saliva (*n* = 7, 50%, respectively) were analyzed for HPV positivity and genotyping. The methods most used for saliva HPV-DNA determination were conventional PCR, nested PCR and quantitative PCR. However, other analytical strategies such as next generation sequencing or immunoassays were also employed for salivary HPV genotyping (Table 1).

### 3.3. Study Quality

Assessment of risk of bias and quality was performed according to NOS (Table S1). Regarding the selection domain, adequate description about characteristics and selection criteria for cases and controls were provided by all of the included studies. Regarding the comparability domain, six out of the 14 studies matched for age and at least one additional factor. Insofar as the exposure domain, few studies reported the blinding of analyses or non-response rates. The mean NOS score in our meta-analysis was six.

### 3.4. Meta-Analysis

#### 3.4.1. Salivary HPV Association with Oral and Oropharyngeal Cancer

**Figure 2:**
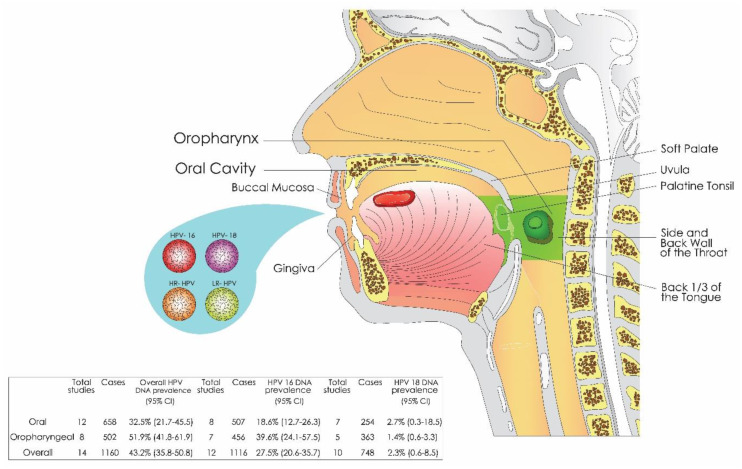
Schematic drawing of salivary HPV and prevalence of oral and/or oropharyngeal cancer. Oral tissue sheds pathogen-infected cells containing different HPV DNA genotypes (HPV16, HPV18, HR-HPV, and LR-HPV) into saliva (with or without oral rinses). The prevalence of salivary HPV DNA varied according to anatomic tumor location, showing the highest infection rate in oropharyngeal carcinomas. In addition, the type-specific prevalence in saliva was also different according to the anatomic tumor location.

Overall, the prevalence of salivary HPV for oral and oropharyngeal carcinoma was of 43.2% (*n* = 1160) while the infection rate in the healthy control group was of 8.9% (*n* = 2304). Salivary HPV16 was the most common type of HPV DNA positive cases (*n* = 1116), representing 27.5% (Figure 2).

**Figure 3:**
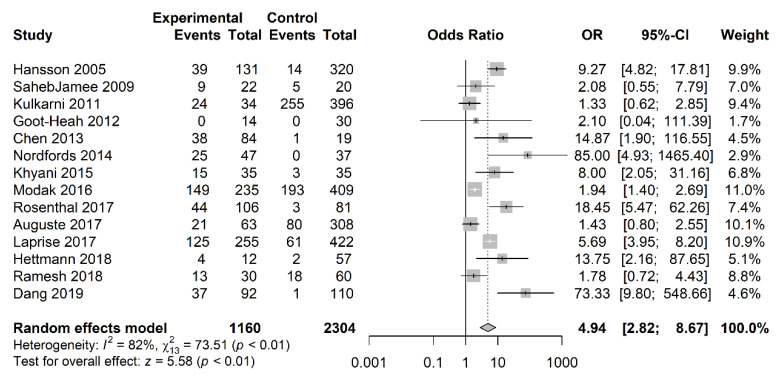
Forest plot for the studies on the association between salivary HPV and oral and oropharyngeal cancer. The squares indicate the ORs (odds ratios) in each study, with square sizes inversely proportional to the standard error of the OR. The diamond shape indicates the pooled ORs. Horizontal lines represent 95% CIs (confidence intervals), I2 > 50% indicates severe heterogeneity.

Our meta-analysis included a total of 1160 cases and 2304 controls. The pooled analysis showed a significant association between positive salivary HPV DNA status and oral and oropharyngeal cancer with a pooled OR of 4.94 (95% CI = 2.82−8.67; *p* < 0.01) (Figure 3). 

**Figure 4:**
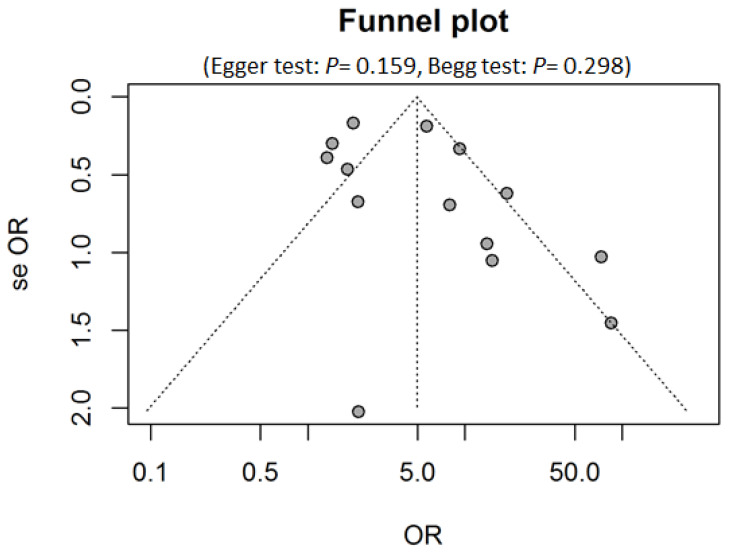
Funnel plot for studies (of 14 studies) on the association between salivary HPV and oral and oropharyngeal cancer. The vertical line represents the pooled OR using random-effect meta-analysis. Two diagonal lines represent (pseudo) 95% confidence limits around the OR for each standard error on the vertical axis. In the absence of heterogeneity, 95% of the studies should lie within the funnel defined by these diagonal lines. Abbreviations: se OR, standard error of odds ratio.

A random-effects model was used because heterogeneity was identified among the 14 studies (I2 = 82%). Visual inspection of the funnel plot revealed a symmetrical (Egger’s test, *p* = 0.159; Begg’s test, *p* = 0.298) distribution of the studies, indicating no evidence of publication bias (Figure 4). 

**Figure 5:**
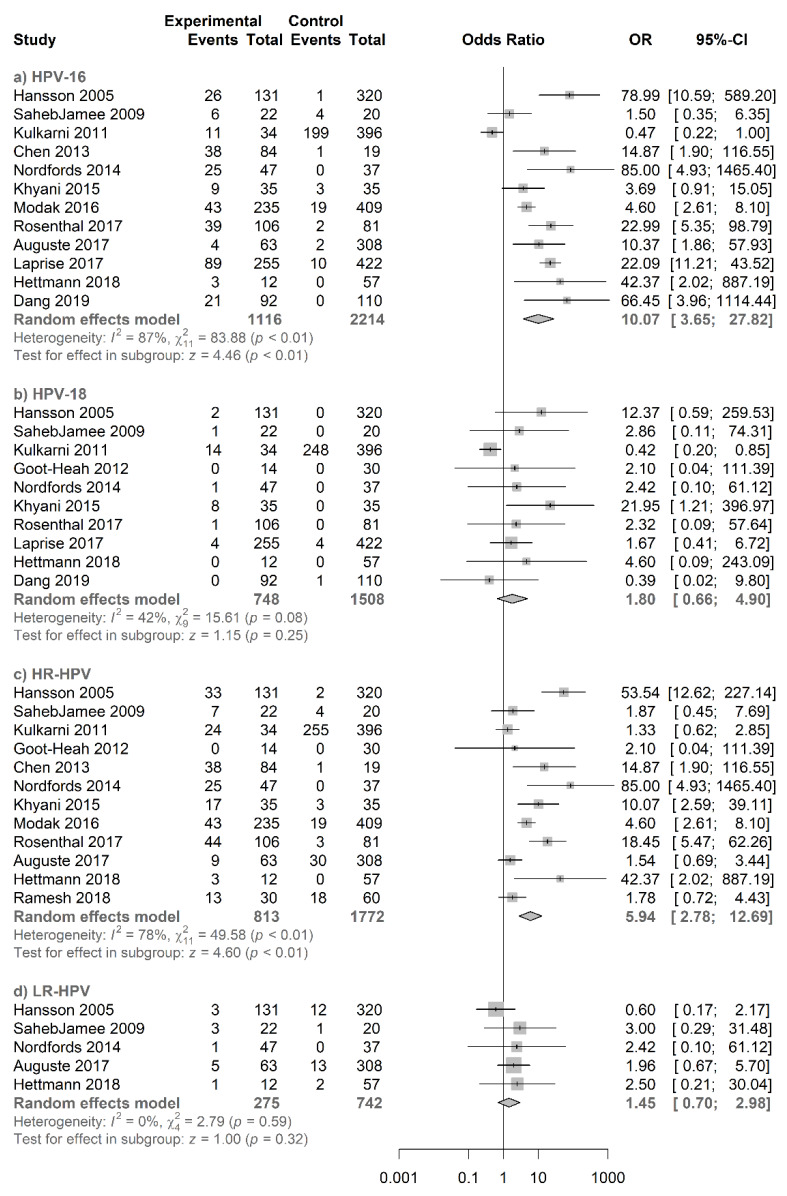
Forest plot for the studies on the association between salivary HPV and oral and oropharyngeal cancer. The squares indicate the ORs in each study, with square sizes inversely proportional to the standard error of the OR. The diamond shape indicates the pooled ORs. Horizontal lines represent 95% CIs. I2 > 50% indicates severe heterogeneity. (**a**) HPV16, (**b**) HPV18, (**c**) HR-HPV, and (**d**) LR-HPV.

For the type-specific analysis (Figure 5), salivary HPV16 showed a significant association with a pooled OR of 10.07 (95% CI = 3.65−27.82; *p* < 0.01). However, salivary HPV18 did not show any significant increased risk for oral and oropharyngeal cancer with a pooled OR of 1.80 (95% CI = 0.66−4.90). In addition, a significant association was found for salivary HR-HPV with OR of 5.94 (95% CI = 2.78−12.69; *p* < 0.01), whereas salivary LR-HPV did not show any significant increased risk with OR of 1.45 (95% CI = 0.70−2.98). The respective funnel plots are represented in Figures S1–S4.

#### 3.4.2. Type-Specific Salivary HPV Association with Oropharyngeal Cancer

**Figure 6:**
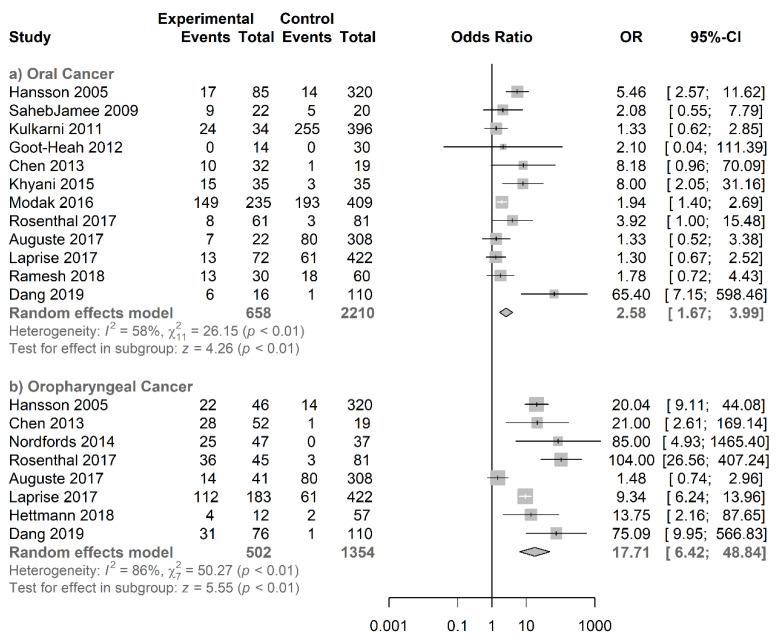
Forest plot for the studies on the association between salivary HPV and anatomic tumor subsites. The squares indicate the ORs in each study, with square sizes inversely proportional to the standard error of the OR. The diamond shape indicates the pooled ORs. Horizontal lines represent 95% CIs. I2 > 50% indicates severe heterogeneity. (**a**) Oral Cancer and (**b**) Oropharyngeal Cancer.

Our subgroup meta-analysis consisted of eight studies, including 502 cases and 1354 controls. In the pooled analysis, salivary HPV DNA infection and oropharyngeal cancer showed a significant association with a pooled OR of 17.71 (95% CI = 6.42−48.84; *p* < 0.01) (Figure 6). 

**Figure 7:**
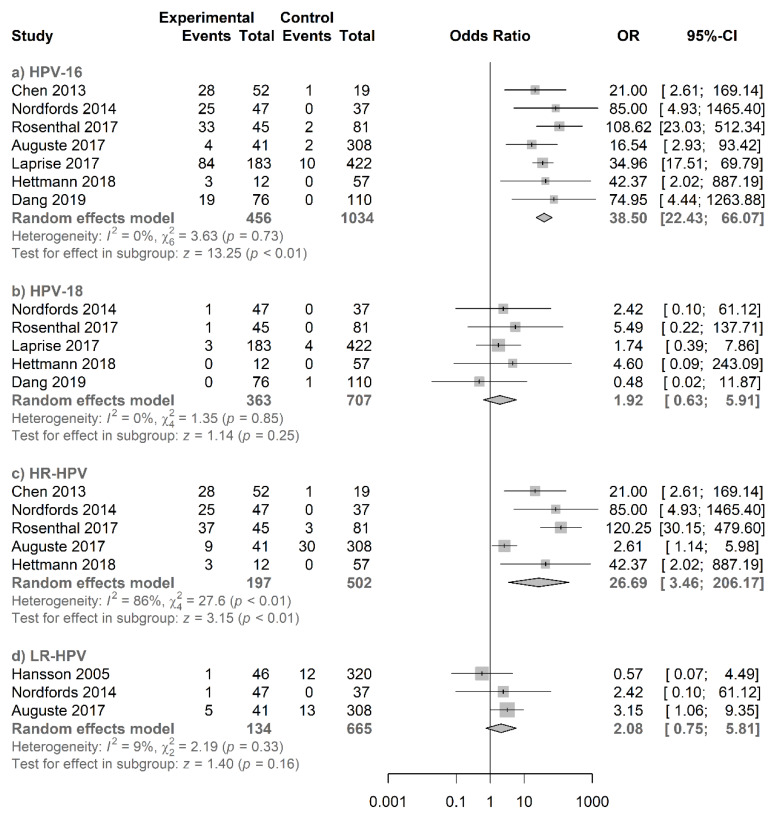
Forest plot for the studies on the association between salivary HPV and oropharyngeal cancer. The squares indicate the ORs in each study, with square sizes inversely proportional to the standard error of the OR. The diamond shape indicates the pooled ORs. Horizontal lines represent 95% CIs. I2 > 50% indicates severe heterogeneity. (**a**) HPV16, (**b**) HPV18, (**c**) HR-HPV, and (**d**) LR-HPV.

According to type-specific analysis (Figure 7), salivary HPV16 showed a significant association with a pooled OR of 38.50 (95% CI = 22.43−66.07; *p* < 0.01) whereas salivary HPV18 showed no significant association with a pooled OR of 1.92 (95% CI = 0.63−5.91). In addition, a significant association was found for salivary HR-HPV with a pooled OR of 26.69 (95% CI = 3.46−206.17; *p* < 0.01) whereas no significant association was found for salivary LR-HPV with a pooled OR of 2.08 (95% CI = 0.75−5.81). Their respective funnel plots are shown in Figures S5–S9.

#### 3.4.3. Type-Specific Salivary HPV Association with Oral Cancer

**Figure 8:**
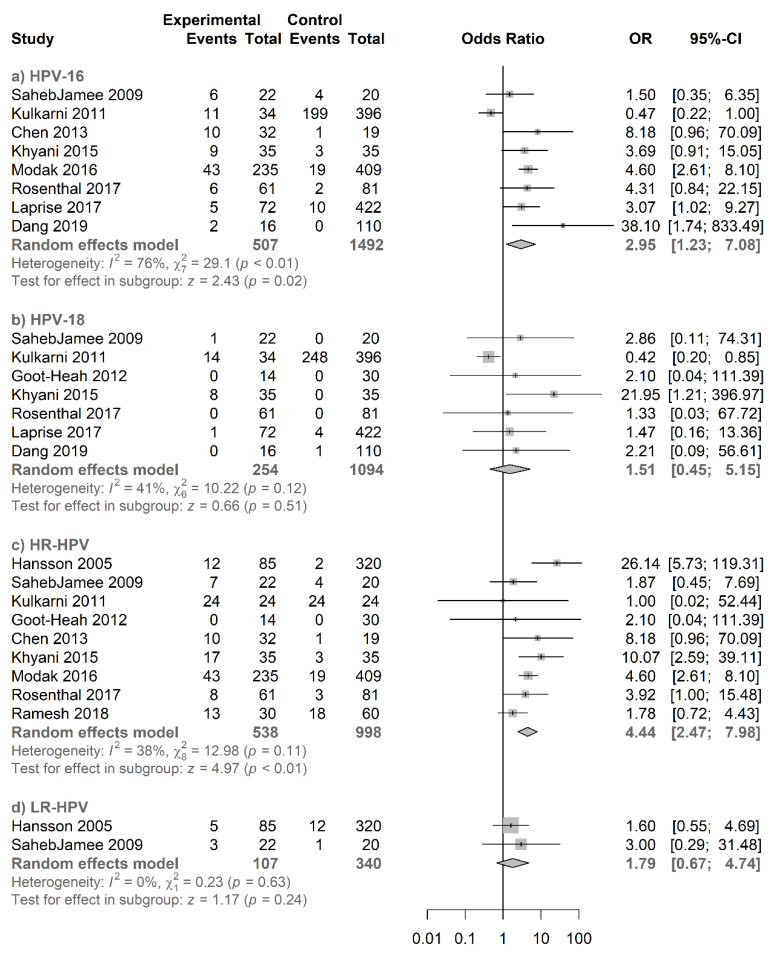
Forest plot for the studies on the association between salivary HPV and oral cancer. The squares indicate the ORs in each study, with square sizes inversely proportional to the standard error of the OR. The diamond shape indicates the pooled ORs. Horizontal lines represent 95% CIs. I2 > 50% indicates severe heterogeneity. (**a**) HPV16, (**b**) HPV18, (**c**) HR-HPV, and (**d**) LR-HPV.

Our subgroup meta-analysis consisted of 12 studies, including 658 cases and 2210 controls. In the pooled analysis, salivary HPV DNA infection and oral cancer showed a significant association with a pooled OR of 2.58 (95% CI = 1.67−3.99; *p* < 0.01) (Figure 6). According to type-specific analysis (Figure 8), salivary HPV16 showed a significant association with a pooled OR of 2.95 (95% CI = 1.23−7.08; *p* = 0.02), whereas no significant association was observed for salivary HPV18 with a pooled OR of 1.51 (95% CI = 0.45−5.15). In addition, a significant association was observed for salivary HR-HPV with a pooled OR of 4.44 (95% CI = 2.47−7.98; *p* < 0.01). However, salivary LR-HPV did not show any significantly increased risk for oral cancer with OR of 1.79 (95% CI = 0.67−4.74). Their respective funnel plots are shown in Figures S10–S14.

## 4. Discussion

In the present study the overall pooled prevalence of salivary HPV-related to oral and oropharyngeal cancer was 43.2%. Similarly, a meta-analysis based on 11 case-control studies evaluating the HPV infection in oral and oropharyngeal cancer found an HPV DNA prevalence of 39.27% [36]. In terms of anatomic tumor location, we observed the highest prevalence of salivary HPV in oropharyngeal cancer (51.9%), whereas the overall percentage in the oral cavity was 32.5%. Similarly, a large comprehensive meta-analysis based on data from 148 studies estimated a pooled HPV DNA prevalence of 45.8% in oropharynx tumors and 24.5% in oral cavity tumors [37]. Although our meta-analysis did not evaluate HPV DNA prevalence in different oropharynx subsites, evidence shows that HPV is most prevalent in tonsils and base of tongue cancers compared to tumors located in walls of oropharynx, uvula and soft palate [38]. 

Overall, in our study, salivary HPV16 was the most commonly detected oncogenic type, accounting for around 28% of cases. As we expected, salivary HPV16 showed a higher prevalence in oropharyngeal cancer (39.6%) than oral cancer (18.6%), in accordance with previous studies [37,39,40]. In particular, the salivary HPV16 prevalence in our study was slightly higher in oral cancer than reported in a meta-analysis by Nydiae et al. [37] (18.6% vs 14.9%), however, other authors have reported higher rates of HPV16 prevalence in oral carcinoma, ranging from 20% to 50% [41,42,43]. Salivary HPV18 was another oncogenic HPV type commonly evaluated by the included studies. Unlike salivary HPV16, HPV18 positivity was found much less frequently, with an overall prevalence of 2.3%. Salivary HPV18 prevalence was even lower in oropharynx tumors (1.7%) as compared to oral cavity tumors (2.7%). One plausible explanation for the decreased prevalence of salivary HPV18 in both oral and oropharyngeal cancers is its specific tropism for glandular tissue and adenocarcinomas, while most head and neck cancers are predominantly of the squamous cell carcinoma type [44]. In addition, HR-HPV has developed a variety of mechanisms facilitating HPV evasion of recognition and clearance by the host immune system [45], which probably contributes to the different viral persistence in each of the anatomic regions of the head and neck. As in our study, Kreimer et al. [40] and Ndyae et al. [37] also found a low HPV18 prevalence in oropharynx tumors (1% and 0.7%, respectively), however, these studies reported a higher HPV18 prevalence in oral cancer (8% and 5.9%, respectively). These differences could be explained by the effect on HPV prevalence of different covariates such as geographical location, lifestyles (alcohol, tobacco or sexual activity), sample size, types of samples and methods used for HPV detection. 

To the best of our knowledge, this is the first meta-analysis evaluating the association between salivary HPV and oral and/or oropharyngeal cancer. The pooled OR showed that oral and oropharyngeal cancer patients had an almost five-fold higher risk of HPV infection than controls. A previous meta-analysis evaluating the presence of HPV in oral and oropharyngeal cancer detected by different methods (histopathology, serum analysis, and cytopathology using OralCDx or oral swishes) reported a significant association with an OR of 2.82 [36]. Overall, our results indicate that salivary HPV causes a higher risk of oral and oropharyngeal carcinogenesis. In addition, we also conducted different subgroup analysis to evaluate the impact of HPV infection on cancer risk according to anatomic tumor location and HPV genotypes. We stratified the salivary HPV studies by anatomical location observing a stronger association between salivary HPV and oropharynx tumors compared to oral cavity tumors. Similarly, Shaik et al. performed a comprehensive metanalysis of HPV-associated head and neck cancers, reporting the highest association for oropharyngeal cancer, with an OR of 14.66, whereas oral cavity and laryngeal cancers had ORs of 4.06 and 3.23, respectively [46]. In addition, we evaluated type-specific salivary HPV risk associated with oral and oropharyngeal cancer. Compared to oncogenic potential, salivary HR-HPV types were associated with an increased risk of oral and oropharyngeal carcinomas. Thus, salivary HPV16 was significantly associated with oral cancer, confirming the findings reported in previous studies [43,47]. Like our study, Hobbs et al. reported a weak statistically significant association between HPV16 and oral cancer, with an OR of 2.0 [45]. On the contrary, a higher association (OR = 9) was reported by Zhu et al., suggesting the potential oncogenic role of HPV16 in oral carcinogenesis in Chinese population [43]. However, in our study, a stronger association was found between salivary HPV16 and oropharyngeal cancer, presenting an OR of 38.50, which suggests the role of HPV16 in the etiology of oropharyngeal cancer. Unlike other meta-analysis [47,48], our study did not analyze association based on specific subsites of the oropharynx. In this sense, a consistent association between HPV16 infection and tonsil cancer has previously been described [47], which seems to indicate a different oncogenic role for HPV infection in the different subsites of oropharynx. 

All the studies included in the present meta-analysis addressed HPV status in oral exfoliated cells collected from saliva with or without oral rinses. In this sense, the first association between oral HPV and oral cancer was reported by Smith et al. [49]. These authors evaluated HPV status in oral exfoliated cells collected by oral rinses from 93 patients and 205 controls finding significantly increased risk (OR = 3.70) of cancer in positive oral HPV patients regardless of alcohol and tobacco use [49]. According to the evidence, salivary HPV DNA represents a promising approach for identifying oral HPV infection. Several authors have shown a significant correlation between HPV DNA detected in tissue and positivity for HPV DNA in saliva, suggesting the potential value of this biofluid for detecting HPV and thus predicting HPV-related head and neck carcinomas [1,50]. Furthermore, salivary HPV DNA has demonstrated to be a good marker for detecting HPV in oropharyngeal cancer, as a high agreement between salivary HPV16 DNA infection and tumor p16 expression has been observed [51,52,53]. However, a recent study revealed a lower sensitivity for identifying p16-positive oral cancer patients through salivary HPV, which may indicate a limited involvement of HPV16 in oral carcinogenesis [54]. Interestingly, our study reviewed the different salivary HPV genotypes identified in oral and oropharyngeal carcinomas, providing additional evidence on the co-existence of multiple HPV types during carcinogenesis. In this matter, saliva analysis represents a great opportunity for the identification and characterization of novel HPVs involved in head and neck cancer.

Our study has several strengths. It is the first meta-analysis highlighting the association between salivary HPV infection and oral and/or oropharyngeal cancer. Moreover, we examined both the overall and the specific prevalence of salivary HPV DNA in oral and/or oropharyngeal cancer. In addition, we performed a comprehensive literature review without language restrictions and the results of our study were in concordance with the scientific evidence. However, the present study is not exempt from limitations. Firstly, the studies included in our meta-analysis were heterogeneous, which could be explained by different factors such as ethnicity, sample size, geographic region, anatomic tumor location, method of HPV detection and different HPV genotypes. Although we performed a subgroup analysis by anatomic tumor location and HPV genotypes, we were unable to elucidate the potential sources contributing to this heterogeneity. Secondly, data such as age, smoking, drinking, sexual habits or diet were not provided by the studies in our sample, hampering the assessment of these confounding variables. Thirdly, some studies included in our analysis could be biased due to the fact that cases and controls were not matched for demographic variables such as age, sex and lifestyle habits. In addition, although almost all these studies analyzed HPV16 and HPV18, we observed high variability regarding HPV genotypes and HPV detection methods, which could substantially affect the results of our analysis. 

## 5. Conclusions

To the best of our knowledge, this is the first meta-analysis addressing the association between salivary HPV infection and oral and oropharyngeal carcinoma. The findings of this meta-analysis provide additional evidence that salivary HPV is associated with oral and oropharyngeal cancer, suggesting that salivary HPV infection is a risk factor for oral and oropharyngeal cancer. However, to validate our findings, future research should focus on prospective cohort studies that explore the occurrence of salivary HPV infection in oral and oropharyngeal cancer. In addition, it is necessary to analyze confounding variables that might be associated with an increased risk of HPV infection in oral and oropharyngeal cancer. 

## Figures and Tables

**Table 1:** Characteristics of the 14 case-control studies included in this meta-analysis.

	Country	Tumor Location (*n*)	Type of Sample/Method of Collection	HPV-Positive Cases (n/N)	HPV-Positive Case Types	HPV-Positive Controls (n/N)	HPV-Positive Control Types	HPV Detection Method
Hansson et al.; 2005	Sweden	OC (85) OPC (46)	Oral rinse/7 mL of 0.9% NaCl solution for 30s	39/131	16, 18, 33, 45, 58, 59, 13, 32, 62, 10, 76	14/320	16, 67, 54, 55, 62, 87, 75, 76, RTRX9	Nested PCR (MY09/ MY11 and GP5+/6+ primers) DNA sequencing
SahebJamee et al.; 2009	Iran	OC (22)	Oral rinse/10 mL of normal saline	9/22	16, 18, 6/11	5/20	16, 6/11	PCR (GP5+/ 6+ primers for L1 region)
Kulkarni et al.; 2011	India	OC (34)	Saliva	24/34	16, 18	255/396	16, 18	PCR (16 and 18 specific primers)
Goot-Heah et al.; 2012	India	OC (14)	Saliva	0/14	-	0/30	-	Nested PCR (MY09/11 and GP5+/6+ primers for L1 region)
Chen et al.; 2013	USA	OC (32) OPC (52)	Saliva/Oragene DNA kits (DNA Genotek)	38/84	16	1/19	16	qPCR (specific primers and probe for E6 region of HPV16)
Nordfors et al.; 2014	Sweden	OPC (47)	Oral rinse/15 mL 50% Listerine® (Johnson and Johnson) for 30s	25/47	16, 18, 67, 6, 51	0/37	-	Bead-based multiplex assay on a MagPix instrument (Luminex Corporation), GP5+/6+ primers for the L1 region and specific primers and probe for E6 region of HPV16
Khyani et al.; 2015	Pakistan	OC (35)	Saliva	15/35	16, 18	3/35	16	qPCR using Real-time PCR Kit HPV16/18 Real-TM Quant (Sacace Biotechnologies)
Modak et al.; 2016	India	OC (235)	Saliva	149/235	16	193/409	16	PCR (HPV 16 specific primer)
Rosenthal et al.; 2017	USA	OC (61) OPC (45)	Oral rinse/10 mL of 0.9% NaCl solution for 30s	44/106	16, 18, * HR-HPV other	3/81	16, * HR-HPV other	qPCR from the HPV L1 region (Cobas® HPV Test-Roche Diagnostics)
Auguste et al.; 2017	France	OC (22) OPC (41)	Saliva/Oragene OG-500 kit (DNA Genotek)	21/63	16, 33, 51	80/308	16	PCR (SPF10 primer system for L1 region, INNO-LiPA® HPV Genotyping Extra; Innogenetics)
Laprise et al.; 2017	Canada	OC (72) OPC (183)	Oral rinse/alcohol-based solution for 15–30s	125/255	16, 18, ** HPV α-9 other than HPV16, *** HPV other	61/422	16, 18, ** HPV α-9 other than HPV16, *** HPV other	PCR (MY09/11 primers for HPV) and genotyping by Linear Array assay (Roche Molecular diagnostics)
Hettman et al.; 2018	Hungary	OPC (12)	Unstimulated saliva	4/12	16, 13	2/57	13, 11	PCR (MY09/11 primers for L1 region) Nested PCR (MY09/11 and GP5+/6+ primers for L1 region), sequencing for genotyping
Ramesh et al.; 2018	India	OC (30)	Oral rinse/10mL of 0.9% normal saline	13/30	16, 18	18/60	16, 18	Nested PCR (MY09/ 11 primers for L1 region)
Dang et al.; 2019	USA	OC (16) OPC (76)	Oral rinse/Original Mint Scope® mouthwash or Crest® Alcohol-free mouthwash (Proctor and Gamble) for 30s	37/92	16, NV14.4, NV69.1, NV95	1/110	18	qPCR (HPV16 E7/HPV18 E7 primers and probe) FAP-PCR from the L1 region NGS and Sanger sequencing

Abbreviations: OC, oral cancer; OPC, oropharynx cancer; PCR, polymerase chain reaction; qPCR, quantitative PCR; FAP-PCR, fluorescent arbitrarily primed PCR; NGS, next-generation sequencing; * HR-HPV other: 31, 33, 35, 39, 45, 51, 52, 56, 58, 59, 66, and 68; ** HPV α-9 other than HPV16: 31,33,35,52,58, and 67; *** HPV other: 6, 11, 18, 26, 34, 39, 40, 42, 44, 45, 51, 53, 54, 56, 59, 61, 62, 66, 68, 69, 70, 71, 72, 73, 81, 82, 83, 84, and 89.

## Supplementary Materials

The following are available online at https://www.mdpi.com/2077-0383/9/5/1305/s1, Table S1: The Newcastle–Ottawa Scale (NOS) for assessing the quality of included studies, Figure S1: funnel plot for studies (of 12 studies) on the association between salivary HPV16 and oral and oropharyngeal cancer, Figure S2: funnel plot for studies (of 10 studies) on the association between salivary HPV18 and oral and oropharyngeal cancer, Figure S3: funnel plot for studies (of 12 studies) on the association between salivary HR-HPV and oral and oropharyngeal cancer, Figure S4: funnel plot for studies (of five studies) on the association between salivary LR-HPV and oral and oropharyngeal cancer, Figure S5: funnel plot for studies (of eight studies) on the association between salivary HPV and oropharyngeal cancer, Figure S6: funnel plot for studies (of seven studies) on the association between salivary HPV16 and oropharyngeal cancer, Figure S7: funnel plot for studies (of five studies) on the association between salivary HPV18 and oropharyngeal cancer, Figure S8: funnel plot for studies (of five studies) on the association between salivary HR-HPV and oropharyngeal cancer, Figure S9: funnel plot for studies (of three studies) on the association between salivary LR-HPV and oropharyngeal cancer, Figure S10: funnel plot for studies (of 12 studies) on the association between salivary HPV and oral cancer, Figure S11: funnel plot for studies (of eight studies) on the association between salivary HPV16 and oral cancer, Figure S12: funnel plot for studies (of seven studies) on the association between salivary HPV18 and oral cancer, Figure S13: funnel plot for studies (of nine studies) on the association between salivary HR-HPV and oral cancer, and Figure S14: funnel plot for studies (of two studies) on the association between salivary LR-HPV and oral cancer.
