# Supplementary material for: Association of Salivary Human Papillomavirus Infection and Oral and Oropharyngeal Cancer: A Meta-Analysis
Source: J Clin Med. 2020 May 1;9(5):1305. doi: 10.3390/jcm9051305 (PMC7290699; doi:10.3390/jcm9051305)
Supplement: Supplementary file 1 [file jcm-09-01305-s001.pdf]

Supplementary Material

Table S1. The Newcastle-Ottawa Scale (NOS) for assessing the quality of included studies.

Study	Subject Selection (Max. 4★)	Study Comparability (Max. 2★)	Ascertainment of exposure (Max. 3★)	Final quality assessment
Hansson et al.; 2005	★★★★	★★	★★	8
SahebJamee et al.; 2009	★★★★	★★	★	7
Kulkarni et al.; 2011	★★★★	0	★	5
Goot-Heah et al.; 2012	★★★★	0	★	5
Chen et al.; 2013	★★★★	0	★	5
Nordfors et al.; 2014	★★★★	0	★★	6
Khyani et al.; 2015	★★★★	0	★	5
Modak et al.; 2016	★★★★	★	★	6
Rosenthal et al.; 2017	★★★★	0	★	6
Auguste et al.; 2017	★★★★	★★	★★★	9
Laprise et al.; 2017	★★★★	★★	★★	8
Hettmann et al.; 2018	★★★★	0	★	5
Ramesh et al.; 2018	★★★★	★★	★	7
Dang et al.; 2019	★★★★	0	★★	6

Note: "★" means one point

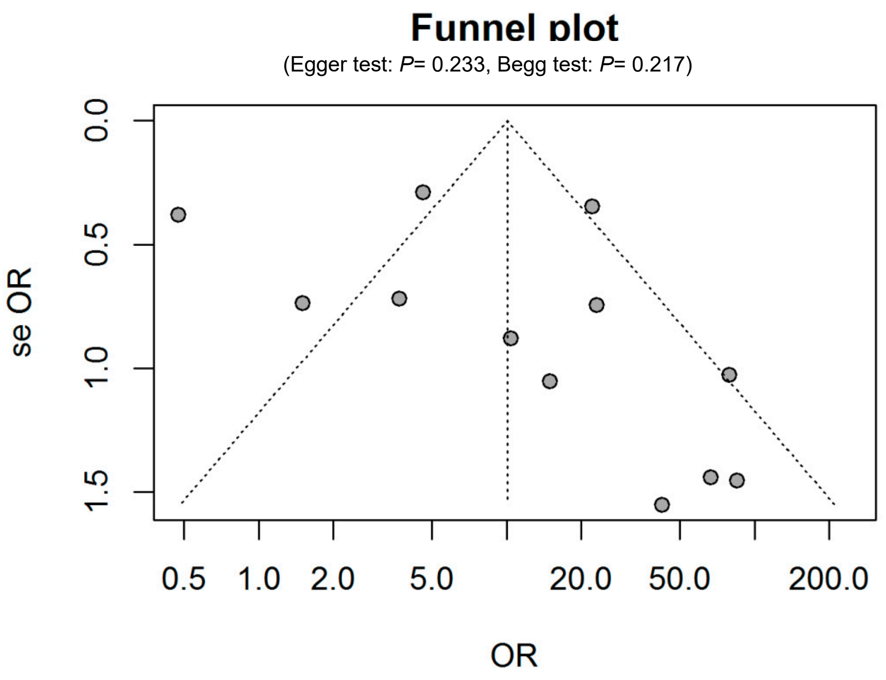

Figure S1. Funnel plot for studies (of 12 studies) on the association between salivary HPV16 and oral and oropharyngeal cancer.

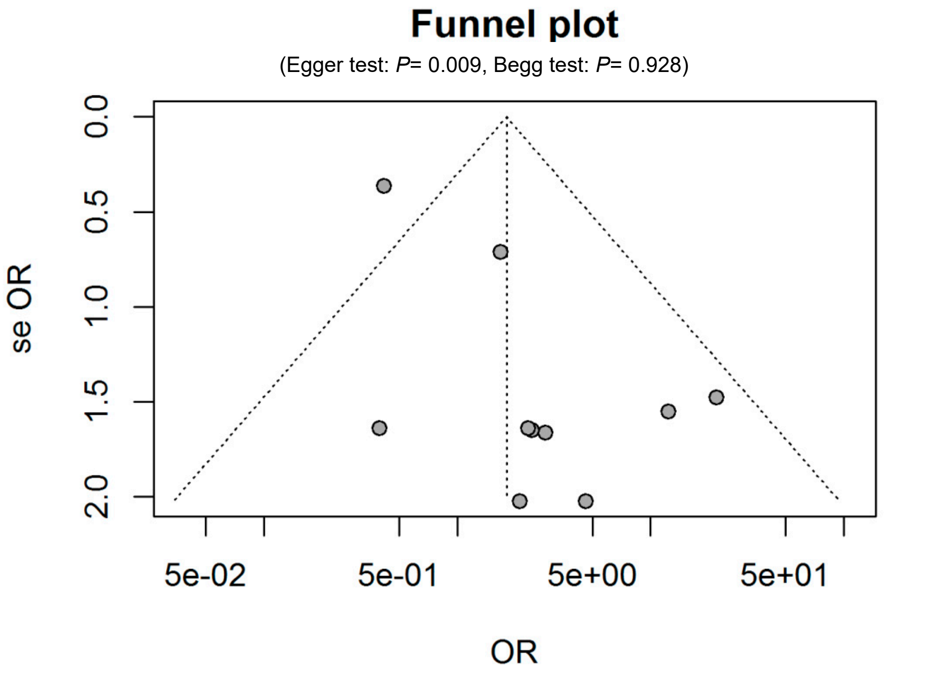

Figure S2. Funnel plot for studies (of 10 studies) on the association between salivary HPV18 and oral and oropharyngeal cancer.

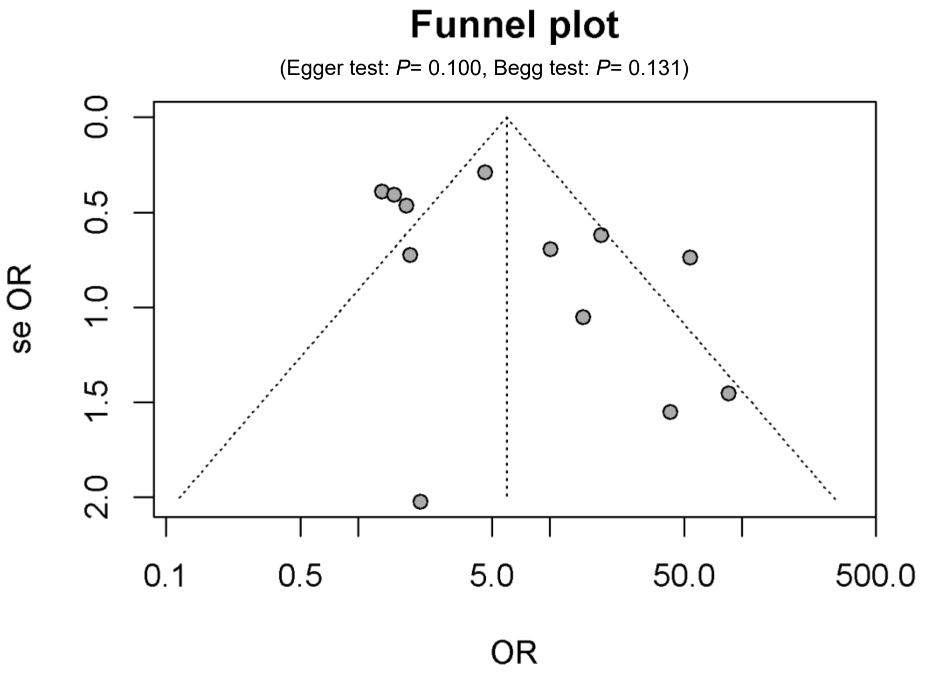

Figure S3. Funnel plot for studies (of 12 studies) on the association between salivary HR-HPV and oral and oropharyngeal cancer.

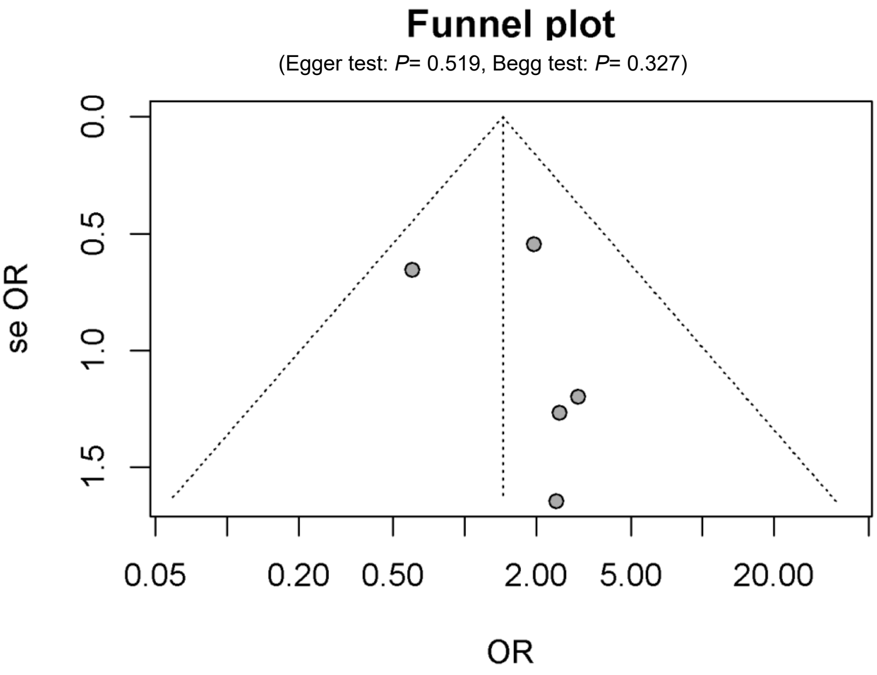

Figure S4. Funnel plot for studies (of 5 studies) on the association between salivary LR-HPV and oral and oropharyngeal cancer.

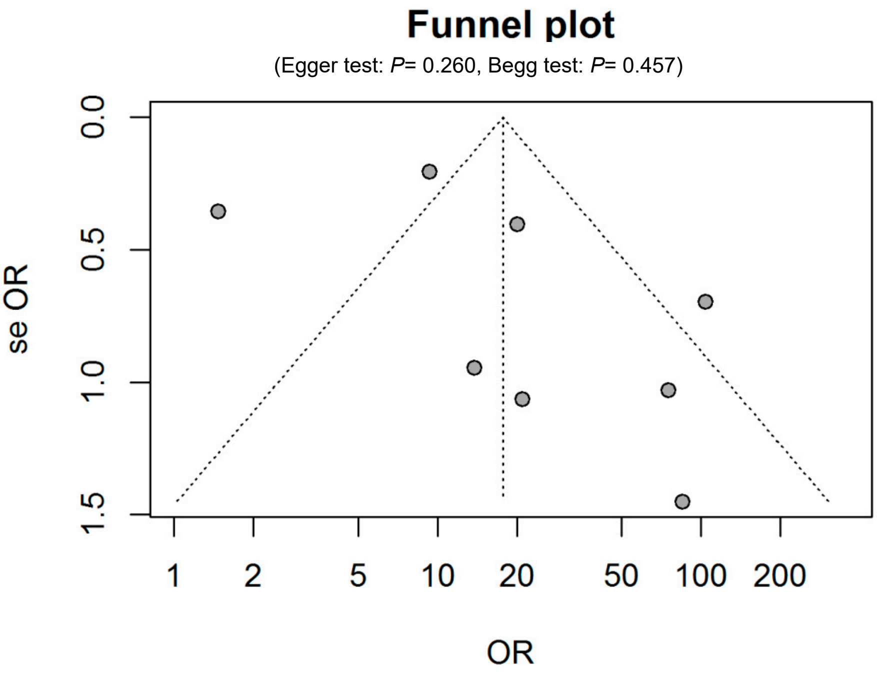

Figure S5. Funnel plot for studies (of 8 studies) on the association between salivary HPV and oropharyngeal cancer.

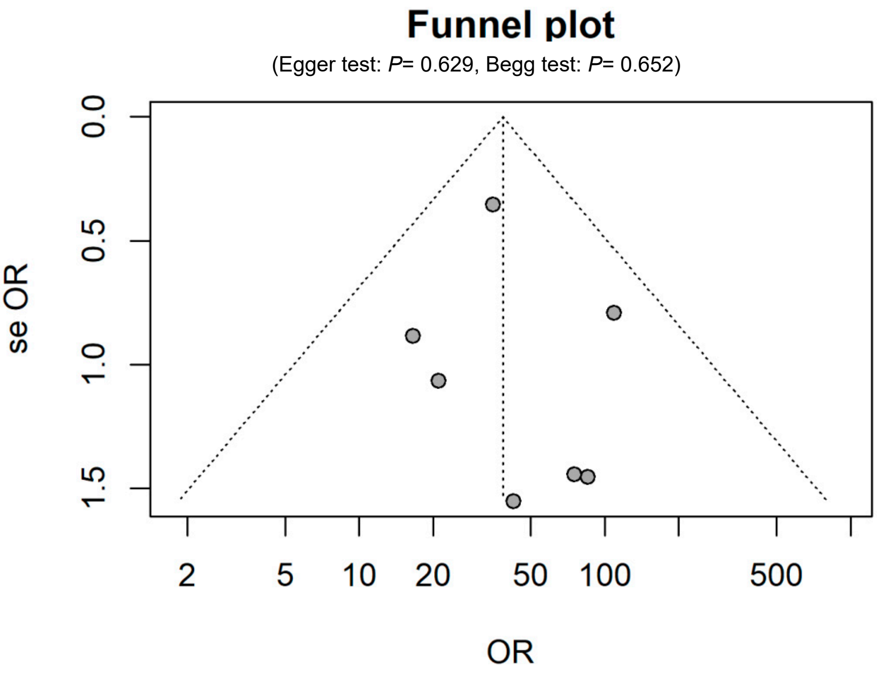

Figure S6. Funnel plot for studies (of 7studies) on the association between salivary HPV16 and oropharyngeal cancer.

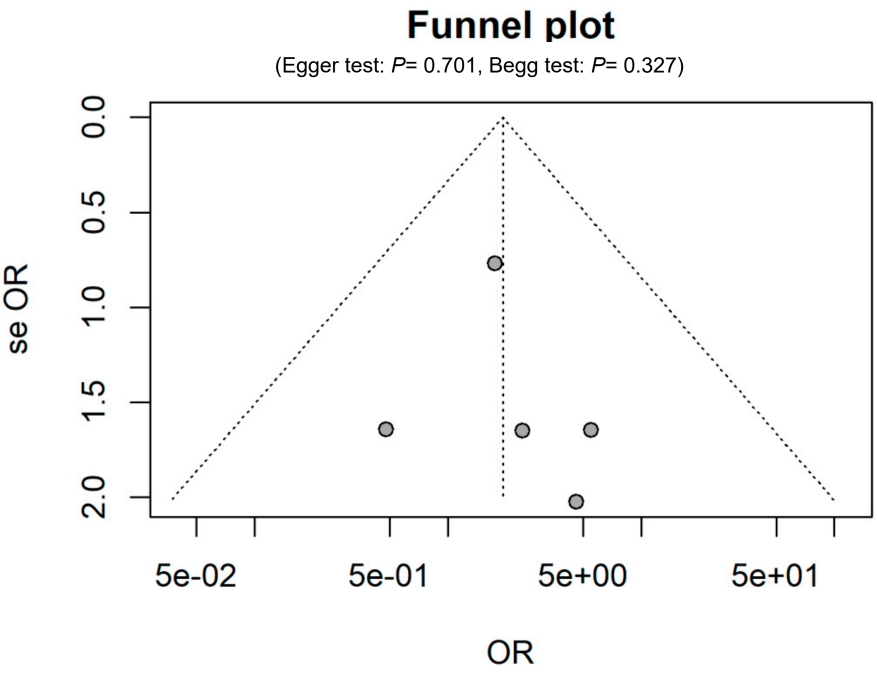

Figure S7. Funnel plot for studies (of 5 studies) on the association between salivary HPV18 and oropharyngeal cancer.

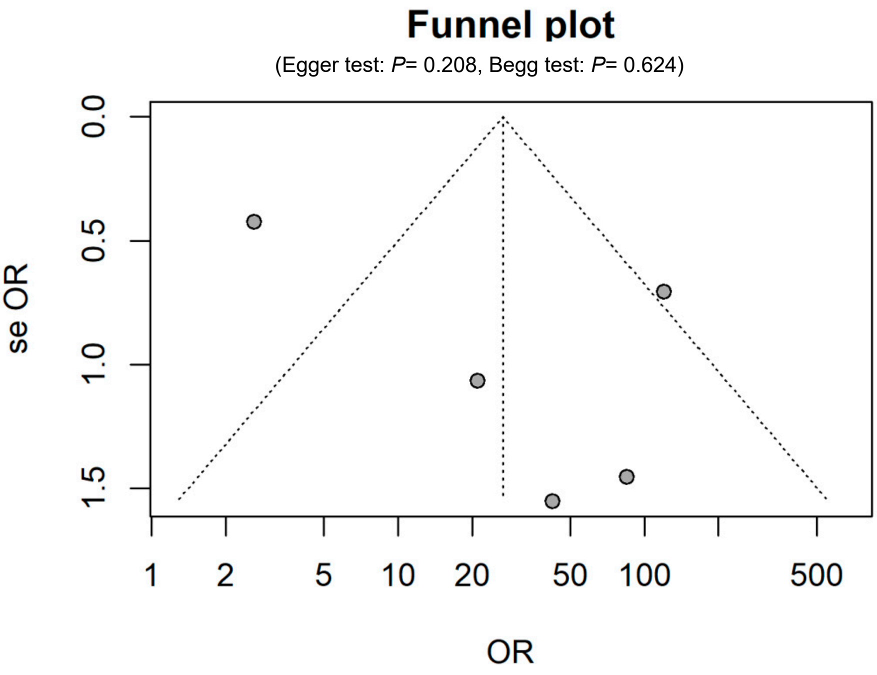

Figure S8. Funnel plot for studies (of 5 studies) on the association between salivary HR-HPV and oropharyngeal cancer.

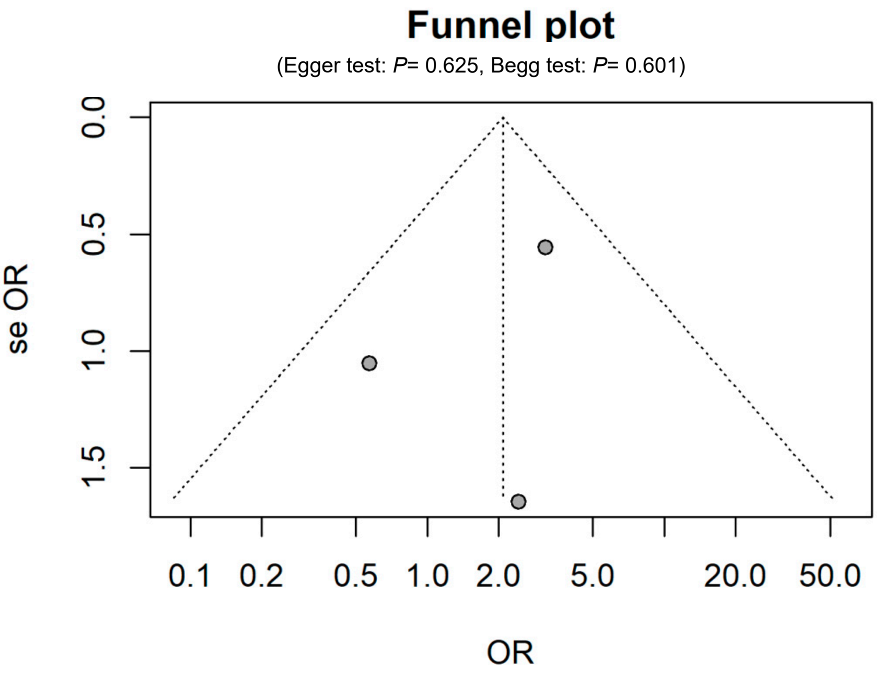

Figure S9. Funnel plot for studies (of 3 studies) on the association between salivary LR-HPV and oropharyngeal cancer.

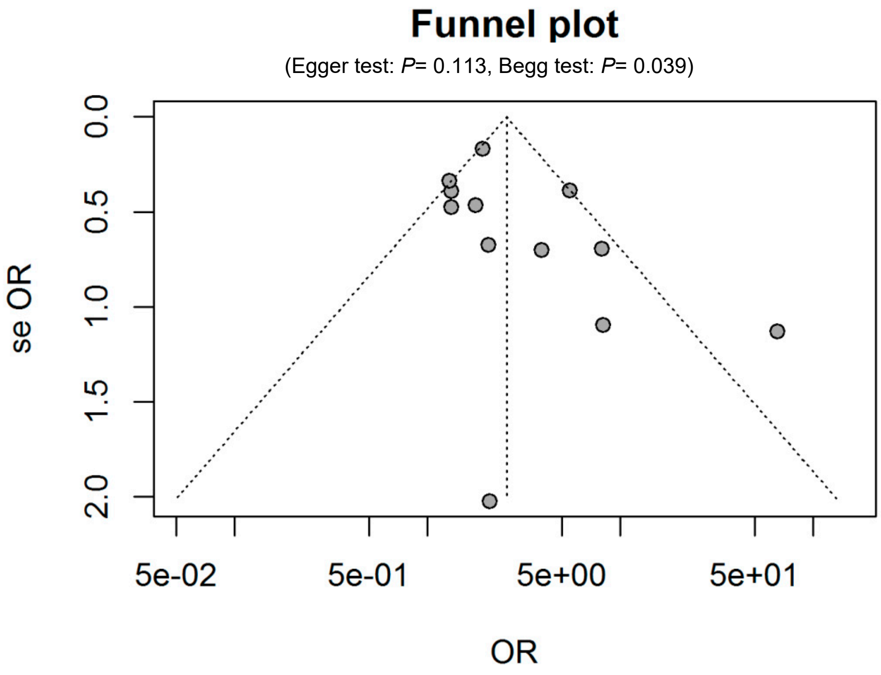

Figure S10. Funnel plot for studies (of 12 studies) on the association between salivary HPV and oral cancer.

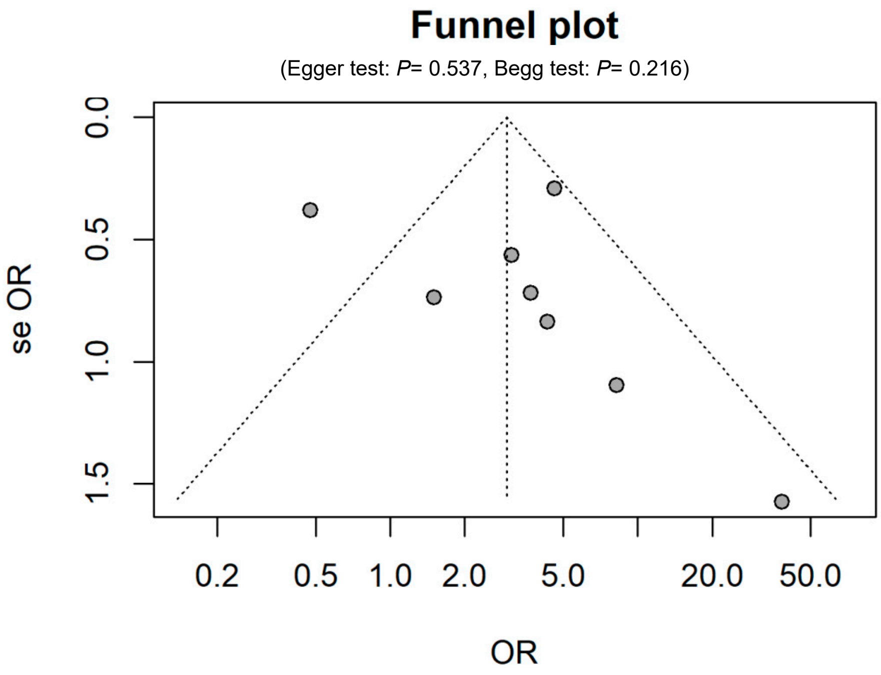

Figure S11. Funnel plot for studies (of 8 studies) on the association between salivary HPV16 and oral cancer.

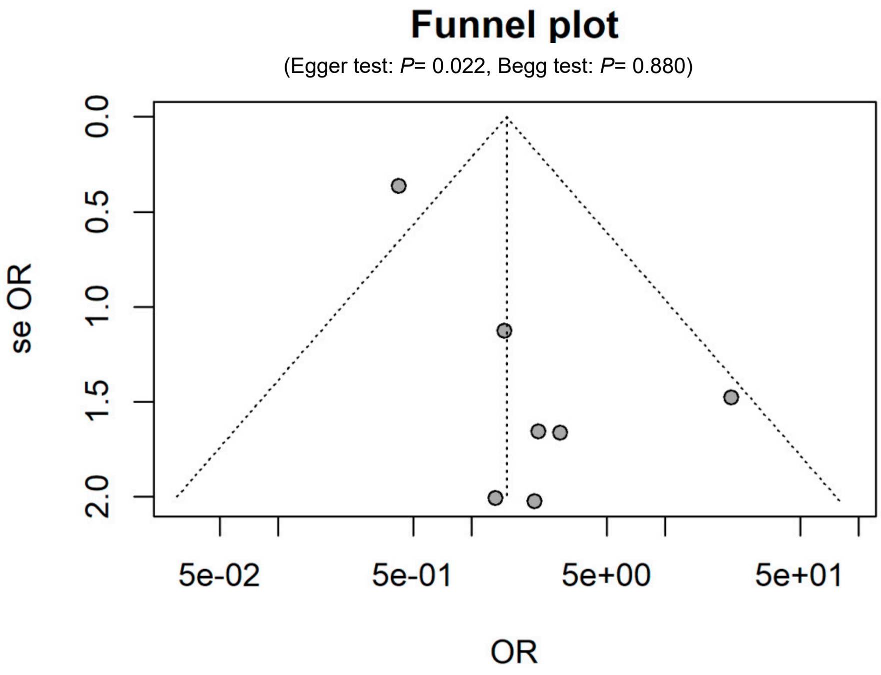

Figure S12. Funnel plot for studies (of 7 studies) on the association between salivary HPV18 and oral cancer.

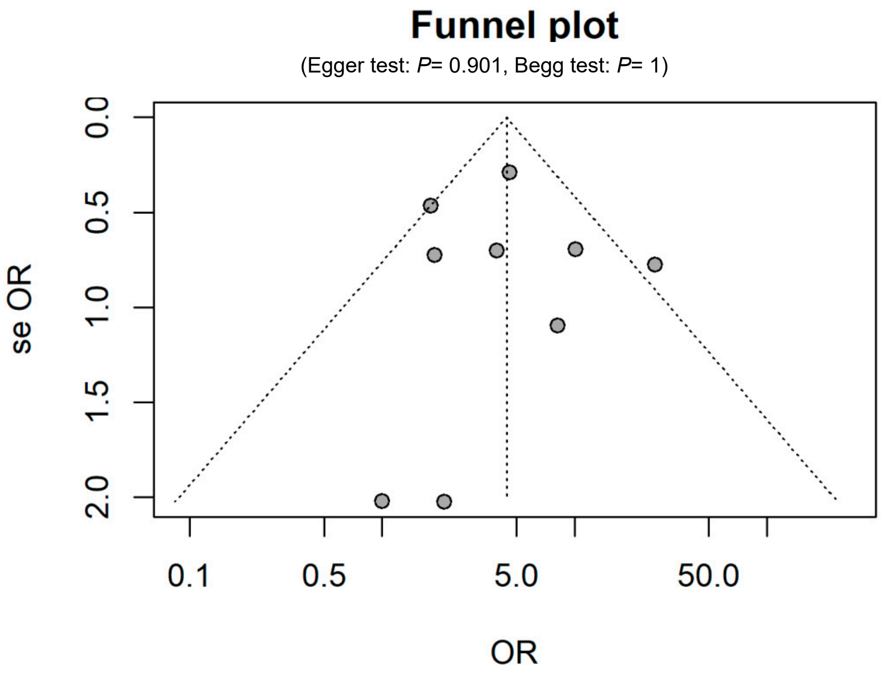

Figure S13. Funnel plot for studies (of 9 studies) on the association between salivary HR-HPV and oral cancer.

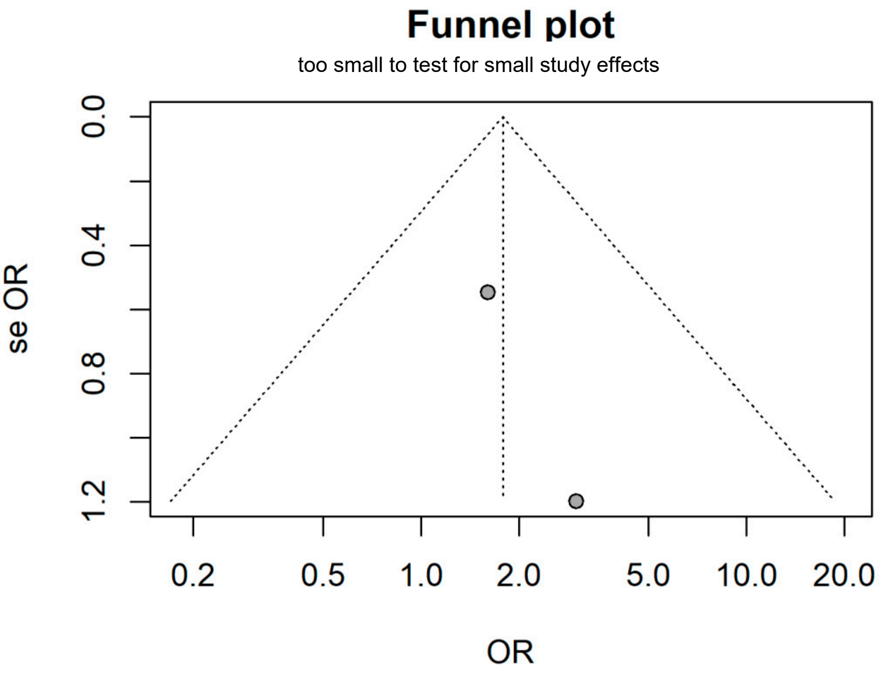

Figure S14. Funnel plot for studies (of 2 studies) on the association between salivary LR-HPV and oral cancer.
